# Enzyme Is the Name—Adapter Is the Game

**DOI:** 10.3390/cells13151249

**Published:** 2024-07-25

**Authors:** Michael Huber, Tilman Brummer

**Affiliations:** 1 Institute of Biochemistry and Molecular Immunology, Medical Faculty, RWTH Aachen University, 52074 Aachen, Germany; 2 Institute of Molecular Medicine and Cell Research, IMMZ, Faculty of Medicine, University of Freiburg, 79104 Freiburg, Germany; 3 German Cancer Consortium (DKTK), Partner Site Freiburg and German Cancer Research Center (DKFZ), 69120 Heidelberg, Germany; 4 Center for Biological Signalling Studies BIOSS, University of Freiburg, 79104 Freiburg, Germany

**Keywords:** cancer, dimerization, ErbB3/HER3, immunodeficiency, inflammatory diseases, KSR1, protein complexes, pseudokinase, SHP2

## Abstract

Signaling proteins in eukaryotes usually comprise a catalytic domain coupled to one or several interaction domains, such as SH2 and SH3 domains. An additional class of proteins critically involved in cellular communication are adapter or scaffold proteins, which fulfill their purely non-enzymatic functions by organizing protein–protein interactions. Intriguingly, certain signaling enzymes, e.g., kinases and phosphatases, have been demonstrated to promote particular cellular functions by means of their interaction domains only. In this review, we will refer to such a function as "the adapter function of an enzyme". Though many stories can be told, we will concentrate on several proteins executing critical adapter functions in cells of the immune system, such as Bruton´s tyrosine kinase (BTK), phosphatidylinositol 3-kinase (PI3K), and SH2-containing inositol phosphatase 1 (SHIP1), as well as in cancer cells, such as proteins of the rat sarcoma/extracellular signal-regulated kinase (RAS/ERK) mitogen-activated protein kinase (MAPK) pathway. We will also discuss how these adaptor functions of enzymes determine or even undermine the efficacy of targeted therapy compounds, such as ATP-competitive kinase inhibitors. Thereby, we are highlighting the need to develop pharmacological approaches, such as proteolysis-targeting chimeras (PROTACs), that eliminate the entire protein, and thus both enzymatic and adapter functions of the signaling protein. We also review how genetic knock-out and knock-in approaches can be leveraged to identify adaptor functions of signaling proteins.

## 1. Introduction

The first cut is the deepest, a first impression is a lasting one, and first thoughts always sneak in again through the backdoor. Imagine someone tells you that Arnold Schwarzenegger is entering the building. Do you think of him as the 38th governor of California or as the Terminator? Either way will make an enormous difference in how you imagine the scene and how you will remember it. The same is true for proteins. If you only know the name “XYZ” of a protein and you are about to hear a talk about its function, most likely you are open-minded for the intriguing function(s) of the protein XYZ the presenter will convey to you in her/his paper. Should you have heard before that XYZ is a kinase, then chances are that you will be biased to hear about data on the kinase activity and possible substrates of the kinase XYZ. This bias will be even more severe should “kinase” be a part of the name of the protein XYZ—well, there has to be a reason why this protein is called “kinase”, right? By focusing on the kinase character of this protein, however, our thoughts and perception of the protein XYZ will almost neglect a different function than “kinase”. Comparable thoughts, of course, apply as well for other signaling proteins carrying an enzymatic function, such as phosphatases, phospholipases, and phosphodiesterases.

Proteins are the central players of our biological world; without them, life would not be possible. Large cellular structures such as the cytoskeleton or the proteasome would not work without proteins (in fact, they are proteins), even though the latter´s task is to degrade proteins. All metabolic processes with their intricate and fine-tuned functions would not even be thinkable without proteins exerting enzymatic functions. Last but not least, cellular communication, signal transduction is a domain of modular protein species exhibiting complex posttranslational and conformational control mechanisms [1]. In signal transduction, proteins are in principle divided into proteins with and without enzymatic function, so-called adaptor or scaffold proteins. Particularly with respect to proteins with enzymatic functions, the latter is often part of the protein´s name and thus, willingly, we tend to connect all the biological functions of this protein with its enzymatic activity. Of course, we do not want to sound like the ones who never got caught in that trap. But we think that it is important to alert students, scientists, and fellow colleagues to the fact that enzymatic activity constitutes only one side, albeit without doubt an important one, of a protein containing one or more catalytic centers.

**Figure 1:**
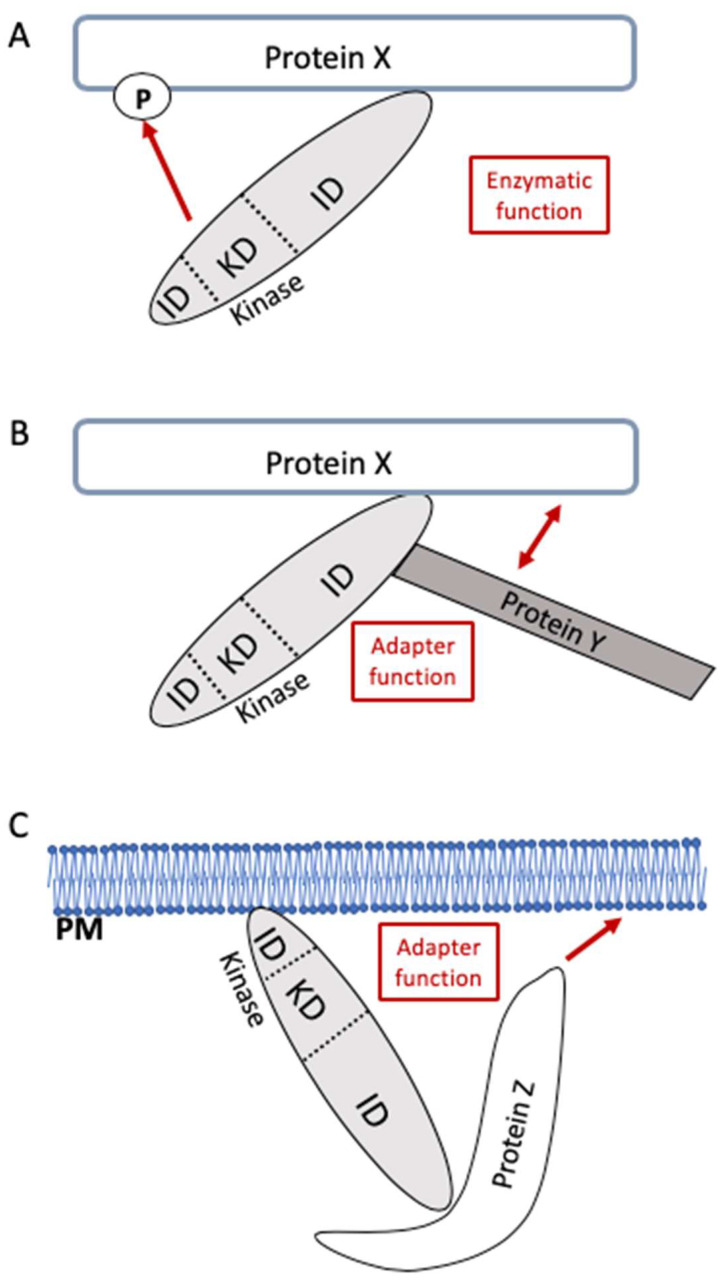
Differentiating between enzymatic function and adapter function. (**A**) An enzyme (in this example, we show a kinase) consisting of interaction domains (ID) and a kinase domain (KD) interacts with protein X in a way that allows the kinase to phosphorylate protein X and modulate its function. We are using the term “enzymatic function” for such interactions. (**B**) The kinase via one of its IDs connects protein X and protein Y, thus allowing these proteins to functionally interact (bilateral arrow). In this example, the enzymatic activity (KD) of the kinase is not relevant for this functional interaction. In such a scenario, we use the term “adapter function”. (**C**) In the situation shown, the kinase interacts with the plasma membrane (PM) or an organelle. Using one of its IDs, the kinase promotes the functional interaction between protein Z and the membrane. In this scenario, the KD of the kinase is not relevant for the function of protein Z (arrow), and we use the term “adapter function” for such situations.

In particular, eukaryotic enzymatic signaling proteins (e.g., kinases, phosphatases, and lipases), which in addition to their catalytic domain can comprise several domains responsible for guiding protein–protein interactions, might be able to perform pure adapter functions as well. Given a kinase (or another type of signaling protein with enzymatic function) via one of its interaction domains binds to another protein (protein X) that subsequently serves as a substrate, then this interaction allows the positioning of the kinase to or at the substrate, and in this review, we will not call this an adapter function of the non-enzymatic part of the kinase (Figure 1A). However, given that the non-enzymatic part of a kinase interacts with another protein X to either allow the connection between protein X and a further protein Y (Figure 1B) or to functionally localize it to a certain region of the cell, such as the plasma membrane or the mitochondria (Figure 1C), then we will refer to this as an adapter function of the kinase protein (independent of its enzymatic activity). Importantly, without this non-enzymatic function of the kinase, protein X would not be able to execute one or more of its functions with respect to cellular signaling or metabolic processes.

Before discussing some intriguing examples of not expected functions of well-known signaling enzymes, we would like to come back to our initial example of Arnold Schwarzenegger and how you imagine his action in a certain situation. Proteins are often named after their enzymatic function, which without doubt is of importance for the protein´s cellular function. However, if the name is “kinase”, then the function of course is “phosphorylation” and most likely this connection will obstruct the view to perhaps many more exciting activities of this protein, which in our example happens to be a kinase. Upon inspection of the primary structure of several kinases, several examples even come to mind where the catalytic domain only occupies a minor part of the polypeptide chain. For example, consider Titin, the largest known protein with more than 3 megadalton, or on a smaller scale the mitogen-activated protein kinase kinase kinase (MEKK) (Supplementary Table S1 in alphabetic order lists all proteins (abbreviations), respective extended names, and functions use in this review) with more than 200 kilodalton (kDa) of which only roughly one quarter is compromised by the kinase domain. In fact, protein serine/threonine kinases like PIM1, PIM2, and PIM3, which are almost exclusively made up by the catalytic domain, rather constitute an exceptional case as almost all kinases contain several protein–protein and/or protein–lipid interaction domains. Indeed, in the next chapters we will concentrate on some kinases and phosphatases and their functions, although non-enzymatic functions have been recently discussed in detail also for proteases cleaving ubiquitin and ubiquitin-like side chains [2] and have emerged for prominent enzymes such as the epigenetic regulator lysine-specific histone demethylase 1A (LSD1) [3]. Thus, as mentioned above, every signaling protein with enzymatic as well as interaction domains might fulfill the statement we would like to promote here: even though the protein has an enzymatic function, exciting stories of the hidden character might be waiting to be told.

## 2. Bruton’s Tyrosine Kinase (BTK) Exerts Important Enzymatic as Well as Adapter Functions in Immune Cells

In a seminal publication, Dr. Ogden C. Bruton reported in 1952 on an immunodeficient boy with a strong disposition for bacterial infections [4,5]. Substitution of gamma-globulin ameliorated the symptoms suggesting that the low levels of gamma-globulin measured in the patient contributed to the disease. About twenty years later, the lack of antibody-producing cells in these patients was shown to be causative for the disease phenotype [6,7]. The disease was named X-linked agammaglobinemia (XLA), and the B lymphocyte development in the bone marrow was compromised at the stage between pro- and pre-B cells [8]. Hence, subsequent developmental stages of B cells are virtually lacking, with respective patients being severely deficient of mature B lymphocytes and plasma cells. The molecular basis of the disease, the lack of the cytoplasmic tyrosine kinase BTK or an expression of a non-functional mutant thereof was independently identified in 1993 by Vetrie et al. as well as by Tsukada et al. [9,10]. BTK-deficiency in mice or expression of the BTK^R28C^ mutant in the CBA/N mouse strain present as milder phenotypes (so-called x-linked immunodeficiency [xid]) compared to XLA in humans [11]. Ablation of BTK expression in mice resulted in reduced numbers of mature conventional B lymphocytes, severe B1 cell deficiency, serum IgM and IgG3 deficiency, and defective responses in vivo to immunization with thymus-independent type II antigens [12]. Again, demonstrating the significant difference between human and murine B cell development and the role of BTK therein, mutation of R28 in human BTK resulted in pronounced XLA immunodeficiency [13].

**Figure 2:**
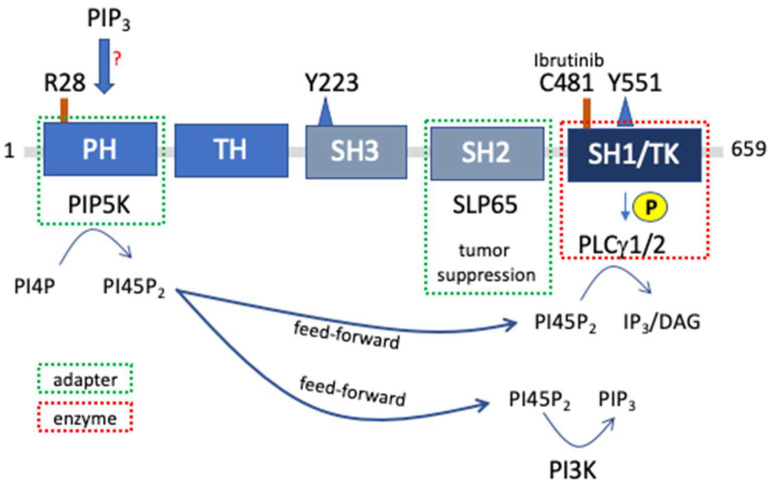
BTK structure and catalytic as well as adapter functions. BTK comprises (from N- to C-terminus) a PH-domain, a TH-domain, an SH3-domain, an SH2-domain, as well as a tyrosine kinase (TK) domain (also known as SH1-domain). The following amino acids are accentuated: Y551 has to be phosphorylated for BTK activation; Y223 can be auto-phosphorylated by active BTK; C481 in the catalytic domain can form a covalent bond with the inhibitor Ibrutinib; and R28 within the PH-domain is crucial for PIP_3_ interaction. The red question mark alludes to the fact that in certain cells upon differential stimulation, BTK activation does not appear to be dependent on PI3K activation (see text). One dominant catalytic function (highlighted by a red dashed rectangle) is the phosphorylation/activation of PLCγ1/2, which then hydrolyzes PI45P_2_ to yield IP_3_ and DAG to promote signaling towards Ca^2+^ mobilization and PKC activation, respectively. The adapter functions known so far (highlighted by green dashed rectangles) comprise the SH2-mediated interaction with the adapter protein SLP65, which together execute a tumor suppressing function in the B-cell lineage, as well as the interaction of PIP5K with BTK’s PH-domain allowing PIP5K translocation to the plasma membrane to phosphorylate the phospholipid PI4P to yield PI45P_2_. BTK-promoted PI45P_2_ production is thought to constitute two feed-forward loops to provide the substrate for both PLCγ1/2 and PI3K.

Structurally, BTK comprises an N-terminal PH-domain (containing the above-mentioned amino acid residue R28), followed by a TH-domain, an SH3-domain, an SH2-domain, and a C-terminal tyrosine kinase domain (Figure 2) [14]. In the course of pre-B and B cell receptor signaling, BTK was demonstrated to be important for tyrosine phosphorylation and activation of phospholipase C-γ2 (PLCγ2) [15] that provides the second messengers, inositol-1,4,5-trisphosphate (IP_3_) and diacylglycerol (DAG), via hydrolyzing phosphatidylinositol-4,5-bisphosphate (PI45P_2_) (Figure 2). This molecular scenario resembles the one depicted in Figure 1A. IP_3_ and DAG regulate the mobilization of intracellular Ca^2+^ and the activation of PKC, respectively, which appear to be crucial signaling events controlling the differentiation and growth of pre-B cells as well as the activation of mature B lymphocytes. Structural connection between BTK and PLCγ2 is mediated by the cytosolic adapter protein SLP65 (also known as B-cell linker protein BLNK), which brings BTK and PLCγ2 in close proximity [15]. Due to the focus on BTK´s catalytic function, the fact that BTK is a PLCγ2 kinase, and the doubtless central role of PLCγ2 for pre-B and B cell receptor function, BTK was mostly, if not only, thought of as a phosphorylating enzyme that additionally contains several interaction domains.

Though BTK deficiency manifests with different severity in humans and mice, the latter offer a valuable model for genetical manipulation to interrogate the role of differential domains of BTK in vivo. Hence, Hendriks and coworkers addressed the role of the catalytic function of BTK by generating transgenic mice that express the kinase-inactive mutant (BTK^K430R^) of human BTK in a *Btk*-deficient background [16] (Table 1). Intriguingly, transgenic expression of the kinase-inactive BTK mutant completely reconstituted usage of λ light chain in BTK-deficient mice [16]. In contrast but still remarkable, defective modulation of pre-B cell surface markers, peripheral B cell survival, and B-cell antigen receptor (BCR)-induced nuclear factor-kappa B (NFκB) activation were partially corrected by expression of the kinase-inactive BTK mutant [16]. Moreover, reconstitution of BTK-deficient chicken DT40 B cells with a kinase-inactive BTK mutant resulted in weak but significant BCR-mediated Ca^2+^ mobilization [17]. Furthermore, overexpression of wild-type or kinase-inactive BTK in the human A20 B cell line resulted in comparable levels of Ca^2+^ mobilization upon BCR triggering [18]. These data indicated that, independent of its kinase activity, BTK can partially function as an adapter molecule during B cell development.

Analyzing B cell development in SLP65-deficient mice, Jumaa and coworkers identified a tumor-suppressing role of SLP65, which was shown to promote differentiation and hence limit pre-B cell expansion in mice [19]. In this line, loss or reduction of SLP65 expression was found in various cases of childhood pre-B acute lymphoblastic leukemia [20]. Intriguingly, BTK was demonstrated to cooperate with SLP65 as a tumor suppressor, and this function was shown to be independent of its catalytic activity, again pointing to its potential adaptor function (Figure 2) [21,22].

Even though the exact molecular basis for BTK’s adapter function in B cell development has not been elucidated until now, an interesting study by Saito et al. might have shed light into this process [18]. They found that BTK via its PH-domain directly interacts with phosphatidylinositol-4-phosphate 5-kinases (PIP5Ks), the enzymes that generate the phospholipid PI45P_2_, which in turn serves as the substrate for PLCγ2 (Figure 2). Hence, BTK not only activates PLCγ2 by means of its enzymatic activity, but also appears to be necessary for BCR-stimulated PI45P_2_ synthesis owing to its adapter function. PI45P_2_ not only is the substrate for PLCγ2 but can also be phosphorylated at its 3′-position by phosphatidylinositol 3-kinases (PI3Ks) yielding phosphatidylinositol-3,4,5-trisphosphate (PIP_3_), a phospholipid described to attract certain proteins containing PH-domains, amongst them BTK. Interestingly, BTK^R28C^, the BTK mutant expressed in the Xid mouse, was demonstrated to have lost its PIP_3_-binding ability and hence could not translocate to the plasma membrane [23]. Thus, compared to wild-type BTK, BTK^R28C^ was unable to transport PIP5K to the membrane, although the underlying BTK^R28C^/PIP5K interaction was still intact (the molecular scenario resembles the one depicted in Figure 1C) [18]. PI3K-dependence of BTK function, however, is not supported by other authors (Figure 2). For instance, no negative influence on BTK activation of PI3K deficiency or use of PI3K inhibitors were observed in B-lymphocytes in a thorough study by Suzuki et al. [24]. Moreover, antigen-triggered activating phosphorylation of BTK as well as of PLCγ1 was independent of PI3K activity in mast cells, though PLCγ1 phosphorylation clearly was dependent on BTK [25,26]. Of note, further data compellingly revealed that BTK can be activated by soluble inositol hexakisphosphate (IP_6_) [27]. IP_6_ induced transient BTK dimerization and thus promoted trans-phosphorylation of the kinase domains. Moreover, generation of IP6 emanates from the production of IP_3_, which at least in mast cells is catalyzed by PLCγ in a PI3K-independent manner. Hence, by both its catalytic and its adapter function, BTK can boost PLCγ-mediated signaling pathways, promoting preB cell differentiation and mature B cell activation. The non-catalytic functions of BTK might also play an important role for treating B cell malignancies such as chronic lymphocytic leukemia for which BTK inhibitors such as ibrutinib are increasingly prescribed, even in first line now [28]. As this ATP competitive inhibitor obstructs the catalytic cleft by establishing a covalent bond with the Cys481 residue in the active center of BTK but cannot remove the protein, cells treated with this drug can still rely on the adaptor function of BTK. In this regard, even kinase-inactive ibrutinib-resistant BTK mutants, such as BTK^C481F^ and BTK^C481Y^, where the bulky Phe and Tyr side chains sterically block access of ATP to the kinase domain, are able to propagate BCR signaling towards the PLCγ2 and NFκB pathway [29] by leveraging some adaptor function. These mutants were shown to be phosphorylated by SRC family kinases at a critical Tyr residue within the catalytic domain resulting in SH2 domain-mediated interaction with hematopoietic cell kinase (HCK), hence resulting in the activation of HCK. Following, HCK can substitute for BTK in phosphorylating PLCγ2 thus propagating BCR signaling [29].

Since BTK belongs to the group of TEC family kinases, in addition to BTK comprising TEC, ITK, BMX, and TXK, which are structurally rather homologous, it is tempting to speculate that other members of this family do serve as kinase-independent adaptors as well. Indeed, TEC also interacted with PIP5K [18], suggesting cooperation of BTK and TEC concerning PI45P_2_ production in cell types expressing both TEC family members. Moreover, interesting data have been published by Grasis et al. with respect to ITK function in T cells [30]. Using murine *Itk*-/- T cells, significant shortcomings in TCR/CD3-triggered actin polymerization were measured. In the Jurkat T cell line, overexpression of wild-type ITK stimulated the endocytosis of latex beads coated with the CD3-specific antibody OKT3. Whereas the extend of bead uptake was severely reduced in cells overexpressing the ITK SH2-mutant ITK^R265K^, overexpression of the kinase-dead mutant ITK^K390R^ exerted a comparable stimulating effect as observed with wild-type ITK [30] (Table 1). This suggested that ITK can mediate actin-dependent cytoskeleton rearrangements independent of its catalytic function. Though not reported on so far, due to their structural homologies, the additional TEC family kinases might act as adapters in certain cellular situations as well.

## 3. The p110γ Isotype of Phosphatidylinositol 3-Kinase (PI3K) Enzymatically Controls Chemotaxis and Uses Its Adapter Function for Regulating cAMP Levels

The PI3K pathway is one of the central signaling pathways in eukaryotic cells and regulates processes like proliferation, growth, survival, metabolism, chemotaxis, and production of inflammatory cytokines [31]. Downstream of various types of signaling receptors, PI3Ks of class I are involved in regulating the above-mentioned cellular processes by phosphorylating PI45P_2_ at its 3′-position yielding PIP_3_, which enables different PH-domain-containing proteins docking to the plasma membrane. PI3K class I is divided into class IA (p110α, p110β, and p110δ) and IB (p110γ), participating in signal transduction from tyrosine kinase-controlled receptors such as receptor tyrosine kinases and cytokine receptors and G-protein-coupled receptors, respectively [31]. In addition to the production of PIP_3_ and subsequent activation of the Ser/Thr kinase protein kinase B (PKB), p110γ stimulated via the βγ heterodimer of heterotrimeric G-proteins is also able to activate the mitogen-activated protein kinase kinase (MEK)/ERK pathway according to the molecular mechanism shown in Figure 1A [32,33]. In contrast to p110α and p110β, which are ubiquitously expressed, and p110δ that is predominantly expressed in leukocytes, p110γ is found to be enriched in white blood cells and cardiomyocytes.

Enormous interest in p110γ and respective pharmacological drugs started when neutrophils and macrophages from p110γ-deficient mice were found to exert severe defects in migration towards chemotactic stimuli [34], which are hallmarks in different inflammatory diseases (Table 1). Disturbing however, the hearts of p110γ-deficient mice showed a significantly increased contractility, and these mice were also found to develop severe myocardial damage after transverse aortic constriction [35]. These in vivo contractility studies were supported by in vitro analyses of single adult cardiomyocytes, which displayed enhanced contractility in the absence of p110γ. In addition, treatment of wild-type cardiomyocytes with a PI3K inhibitor caused a significant increase in contractility [35]. To this point, these studies convincingly indicated that it would be detrimental for patients to use p110γ inhibitors, which could alleviate inflammatory occurrences while aggravating heart function.

Intriguingly, a significant increase in the relaxation rate could be measured in p110γ-deficient cardiomyocytes compared to wild-type cells [35], a process known to be modulated by cAMP-dependent signaling pathways [36]. In line, cAMP levels were increased in cardiomyocytes from p110γ-deficient mice and enhanced contractility was attenuated by a cAMP antagonist, suggesting that p110γ controls adenylyl cyclase [35]. The generation and analysis of a mouse carrying a targeted mutation (K833R) in the gene of p110γ, which causes a loss of kinase activity [37], provided interesting answers and allowed this scientific journey to take a new turn. This mouse displayed reduced inflammatory reactions corroborating the role of p110γ in leukocyte migration and inflammation; however, its cAMP levels in cardiomyocytes as well as heart function were comparable to wild-type mice [37]. These data suggested an adapter function of p110γ, affecting either production or hydrolysis of cAMP. Hence, interactions of p110γ with one of the regulatory or catalytic subunits of protein kinase A (PKA), or with one of the cAMP-hydrolyzing phospho-diesterases (PDEs) were amongst the justified possibilities. Indeed, cardiac p110γ could be demonstrated to constitutively interact with PDE3B (the molecular scenario most likely resembles the one depicted in Figure 1B) [37]. Moreover, p110γ was shown to promote PDE3B activity in a kinase-independent manner, proofing an important adaptor function of this lipid kinase. The elucidation of this adaptor function was well received by various pharmaceutical companies developing p110γ inhibitors for use in patients suffering from chronic inflammatory conditions. For sure, the interim status of a detrimental effect of p110γ deficiency (and thus p110γ inhibition) on physiological heart function would not have promoted such a pharmaceutical development.

## 4. The Hemopoietic Lipid Phosphatase SH2-Containing Inositol Phosphatase 1 (SHIP1) Controls RAS Activity by Means of Differential Adapter Functions

In signal transduction, a balanced interplay between positive and negative signals is mandatory for allowing physiological homeostasis. An important PI3K antagonistic activity, particularly in cells of the hemopoietic system is provided by the SH2-containing inositol 5′-phosphatase 1 (SHIP1), which dephosphorylates PIP_3_ to yield PI34P_2_ [38] (Table 1). This contrasts to the inositol 3′-phosphatase and tumor suppressor, PTEN, exactly reverting the PI3K-catalyzed reaction [39]. Hence, SHIP1 not only attenuates cellular PIP_3_ concentration, but also generates the second messenger PI34P_2_, which can bind to the PH-domains of particular signaling/adapter proteins (e.g., BAM32 and TAPP2 [40]).

**Figure 3:**
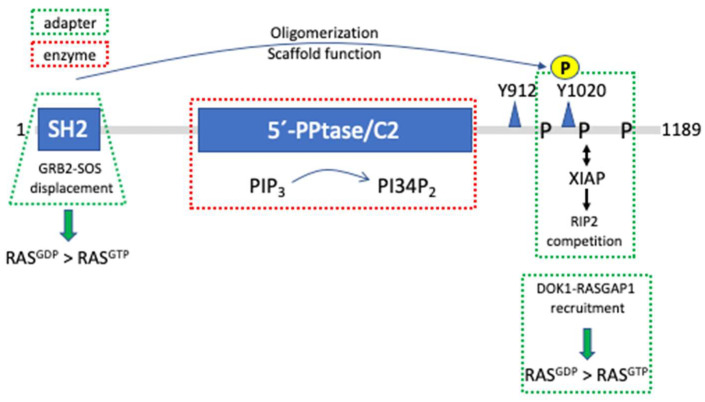
SHIP1 structure and catalytic as well as adapter functions. SHIP1 comprises (from N- to C-terminus) an SH2-domain, a catalytic 5′-phosphatase (PPtase) domain containing a PI34P_2_-binding C2 domain allowing allosteric feed-forward enhancement, and a proline-rich (P) C-terminus additionally comprising two NPxY sequences (Y912 and Y1020). As an enzyme, SHIP1 hydrolyzes the phospholipid PIP3 to yield PI34P_2_ (highlighted by a red dashed rectangle). The adapter functions reported so far (highlighted by green dashed rectangles) comprise the SH2-mediated displacement of the GRB2-SOS complex from the adapter protein SHC, thus repressing GDP-to-GTP exchange at RAS (resulting in enhanced RAS^GDP^ to RAS^GTP^ levels (RAS^GDP^ > RAS^GTP^)) as well as the recruitment of the DOK1-RASGAP1 complex to a C-terminal phosphorylated NPxY sequence via DOK1´s PTB-domain, hence causing enhanced GTP-to-GDP hydrolysis at RAS (RAS^GDP^ > RAS^GTP^). Moreover, the E3 ubiquitin ligase XIAP can interact with the proline-rich tail of SHIP1, thereby displacing XIAP from RIP2, and blocking NFκB activation. Furthermore, the SH2-domain of SHIP1 has been demonstrated to interact with phosphorylated Y1020, allowing dimerization (and most probably oligomerization) of SHIP1 to promote its potential scaffolding function.

SHIP1 is a protein of 145 kDa comprising various domains and interaction sequences [41]. An N-terminal SH2-domain is followed by a central 5′-phosphatase domain, which comprises the catalytic center as well as a C2-domain able to bind PI34P_2_. It has been demonstrated that SHIP1 activity can be allosterically enhanced by the product of its catalytic reaction [42]. In its C-terminus, SHIP1 contains several proline-rich regions of both type I and type II, enabling the phosphatase to interact with various SH3-domain-containing proteins. Moreover, the C-terminus holds two NPxY sequences that when phosphorylated can interact with PTB-domain-containing adapter proteins such as SHC and DOK1 (Figure 3) [43].

Since SHIP1 is a cytosolic protein and its substrate, PIP_3_, is a membrane-bound phospholipid, SHIP1 has to translocate to the membrane in order to be able to fulfill its catalytic function. Thus, SHIP1’s various interaction modules, by binding in a direct or indirect manner to cell surface receptors, are mandatory for igniting its catalytic function (the molecular scenario resembles the one depicted in Figure 1A) with the exception that SHIP1 is not phosphorylating proteins, but dephosphorylating a phospholipid). The SH2-domain of SHIP1, for instance, binds with high affinity to the tyrosine-phosphorylated ITIM of the inhibitory receptor FcγRIIB (CD32B) upon co-crosslinking of the FcγRIIB and the BCR on B-lymphocytes, resulting in the attenuation of PKB and MAPK activation as well as Ca^2+^ mobilization compared to BCR stimulation alone [44,45]. Whereas reduction of PKB activation and Ca^2+^ mobilization was dependent on the catalytic activity of SHIP1, MAPK regulation was found to be dependent on SHIP1’s adaptor function. BCR-mediated RAS and subsequent MAPK activation follows the formation of a protein complex comprising the two adapter proteins SHC and GRB2, and the guanine nucleotide exchange factor SOS [46]. This complex is disintegrated upon BCR/FcγRIIB co-crosslinking, thus abrogating MAPK activation. FcγRIIB-bound SHIP1 is released from the phospho-ITIM by interacting with SHC in a bidentate fashion. Thus, the SH2-domain of the small adapter protein GRB2 bound to SHC is displaced by the SHIP1 SH2-domain, resulting in the attenuation of MAPK activation in a SHIP1-dependent manner, however, independent of SHIP1’s catalytic activity (Figure 3) [47,48]. Corroborating, co-ligation of BCR and FcγRIIB in *Ship1*-/- B lymphocytes does not result in the attenuation of ERK activation [49].

Intriguingly, a second mechanism involving SHIP1’s adapter function in attenuation of ERK activation upon BCR/FcγRIIB co-aggregation has been described in [50] (Table 1). Here, upon co-ligation the adapter protein DOK1, via its PTB-domain interacts with tyrosine-phosphorylated, FcγRIIB-bound SHIP1. Subsequent tyrosine phosphorylation of DOK1 enables interaction with an SH2-domain of the RAS GTPase-activating protein RASGAP1, eventually reducing RAS and subsequent ERK activation (molecular level resembling Figure 1B) (Figure 3). This mechanism has been described for FcεRI/FcγRIIB co-crosslinking in mast cells as well [51]. In conclusion, RAS and subsequent ERK activation as well as inhibition appears to be controlled by SHIP1 independent of its catalytic activity in B cells upon BCR/FcγRIIB co-aggregation.

Though no 3D structure of SHIP1 is available yet, a comparison with its cousin, the inositol polyphosphate 5′-phosphatase Synaptojanin, allows for an interesting suggestion. As for SHIP1, the catalytic domain of Synaptojanin is located centrally in the molecule and both N- and C-termini are entering/leaving the catalytic domain on the same side as demonstrated by the crystal structure, offering the possibility of interaction between both ends of Synaptojanin [52]. Interestingly in this regard, Mukherjee et al. have shown that the SHIP1 SH2-domain can bind to the phosphorylated Y1020 in SHIP1’s C-terminus [53]. In this respect, intermolecular interaction between different SHIP1 proteins could be demonstrated. Although the technique used did not allow a statement about the number of SHIP1 molecules in such a complex, the valid possibility exists that SHIP1 forms oligomers of different sizes, hence expanding its capacities as an adapter/scaffold protein (Figure 3). Particularly, as mentioned before, its C-terminus comprising several proline-rich as well as two NPxY sequences offers the potential for manifold interactions with SH3- and PTB-domain containing proteins.

One very intriguing protein in this respect is the adapter protein SH3 domain-containing kinase-binding protein 1 (also known as CIN85), which is known to be involved in various cellular processes such as signal transduction, cytoskeleton remodeling, and vesicle-mediated transport/endocytosis [54]. CIN85 contains three SH3-domains in its N-terminus and various laboratories have found CIN85 interacting with SHIP1 [55,56,57]. Kühn et al. found that a C-terminal, coiled-coil domain of CIN85 allows for its trimerization, thus resulting in a homomeric protein complex containing nine SH3-domains. In B-lymphocytes, such CIN85 trimer was found to interact with several molecules of the central adapter protein SLP-65, which organizes differential signaling enzymes/pathways upon BCR activation, particularly PLCγ2. SLP-65 proteins were capable of interacting with more than one CIN85 trimer, hence perpetuating the oligomerization process [57]. Given that SHIP1 is also capable of interacting with more than one CIN85 SH3-domain, hetero-oligomerization between SHIP1 and CIN85, amongst others, would be conceivable. Thus, SHIP1 as part of a large adapter complex might be capable of scaffolding various signaling or metabolic functions independent of its phosphatase domain.

An additional function of SHIP1 independent of its catalytic activity has been reported by Conde et al. [58]. The proline-rich C-terminus of SHIP1 was shown to interact with the Bir2 domain of the E3 ubiquitin ligase XIAP, thereby interfering with the interaction of XIAP and the receptor-interacting serine/threonine-protein kinase 2 (RIP2). The XIAP/RIP2 complex is necessary for the NOD2-mediated activation of the NFκB pathway in response to bacterial peptidoglycans. Hence in macrophages SHIP1 is capable of attenuating NOD2-induced NFκB activation by disturbing the interaction of XIAP with RIP2 independent of its phospholipid phosphatase function (Figure 3) [58]. Intriguingly, in addition to and independent of its kinase function, RIP2 participates in LPS-induced signal transduction in macrophages by organizing a protein complex containing IRAK1 and TRAF6 at the LPS receptor TLR4. Analysis of *Rip2*-deficient macrophages revealed the importance of RIP2 for the activation of the NFκB- as well as p38^MAPK^ pathways. Excluding its kinase function, LPS signaling in macrophages expressing a kinase-dead mutant of RIP2 was shown to be intact [59].

## 5. Examples from Receptor Tyrosine Kinase (RTK)-MAPK Pathways

**Figure 4:**
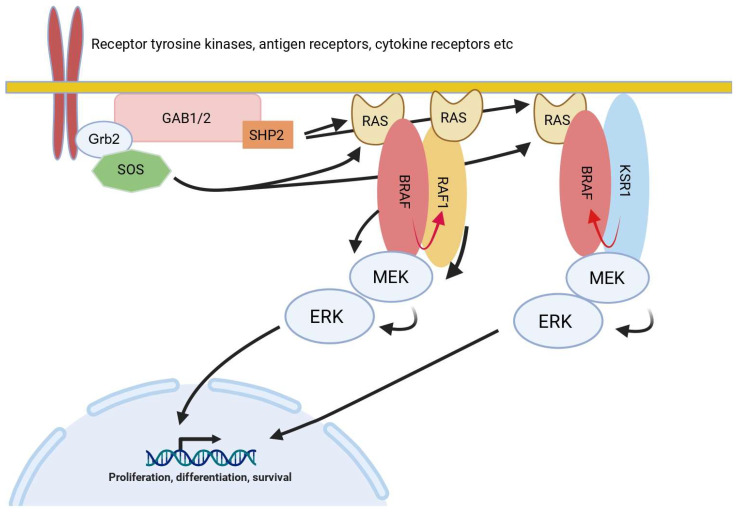
Simplified sketch of the RAS/ERK MAPK pathway. This pathway is activated by a plethora of receptor tyrosine kinases (RTK), such as the subsequently mentioned HER2/HER3 heterodimer, but also antigen and cytokine receptors [61,62,63]. Phosphorylation of the receptor tails, either by intrinsic enzymatic activity as in the case of RTKs, or by associated protein tyrosine kinases of the SRC, SYK, or JAK families in case of antigen and cytokine receptors, results in the recruitment of SH2 domain containing adaptor proteins like GRB2. With its two SH3 domains, GRB2 can then recruit the guanine nucleotide exchange factor SOS and docking proteins of the GAB family, which, upon tyrosine phosphorylation, recruit and allosterically activate SHP2 by engaging with its tandem SH2 domain [64,65]. Both SOS and active SHP2 are required for optimal RAS signaling. Active RAS not only recruits RAF family members, such as BRAF and RAF1 to the plasma membrane, but also induces conformational changes resulting in the exposure of their kinase domains and homo- or heterodimerization, leading to the allosteric transactivation of RAF protomers indicated by the swung red arrow [66,67,68]. Likewise, the pseudokinase KSR1 or RAF proteins rendered inactive either by mutations abolishing enzymatic activity or by ATP competitive kinase inhibitors can also trigger allosteric transactivation of catalytically competent RAFs [69,70]. Figure was created with BioRender.com (accessed on 27 April 2024).

The RTK-MAPK pathways also provide several examples for signaling elements in which the various proteins, in addition to their enzymatic functions, fulfill critical non-catalytic functions (Figure 4, either by containing protein–protein and/or protein–lipid interaction domains or by assuming a pseudoenzyme role [60]. As these pathways receive increasing attention as drug targets, in particular but not limited to the areas of oncology and inflammatory diseases, an in-depth understanding of these non-catalytic function is key to understanding of drug action, including side-effects, and failure, e.g., within the context of drug resistance.

### 5.1. The Pseudokinase HER3/ErbB3 Contributes to Activation of EGFR Family Members in a Kinase-Independent Manner

HER3 is a member of the epidermal growth factor receptor (EGFR) family of RTKs, comprising the catalytically competent members EGFR (ErbB1), HER2 (ErbB2), and HER4 (ErbB4), which play a critical role in normal physiology and ontogeny, but have become important drug targets in oncology, e.g., by serving as targets for small-molecule inhibitors, such as gefitinib, afatinib, or lapatinib, or for therapeutic antibodies such as cetuximab or trastuzumab [61,71]. While EGFR, HER2, and HER4 are catalytically competent, HER3 is considered as a pseudokinase, although it might display residual activity under certain circumstances (as discussed in Ref. [60]). Interestingly, EGFR, HER3, and HER4 are bound by a diverse array of ligands, while HER2 displays relatively high intrinsic activity, but lacks a known ligand. All four EGFR/HER/ErbB family members form homo- and heterodimers. Ironically, the HER2/HER3 heterodimer, in which the “deaf dances with the dumb” [72], unfolds very high biological activity in response to growth factors such as Neuregulin. There are at least two mechanisms that explain this conundrum. First, dimerization of protein kinases often involves conformational changes in the catalytic domains by which one protomer activates another through an allosteric mechanism [73,74] (Table 1). RTKs are no exception and in the case of the HER2/HER3 heterodimer, HER3, once “activated” by Neuregulin binding stimulates HER2 by dimerization. HER2, in turn, phosphorylates tyrosine residues in the cytoplasmic moiety of HER3, which serve as critical PI3K recruitment sites [75,76]. Thus, irrespective of the question of whether HER3 retains intrinsic activity, it serves at least three critical functions for its catalytically active partner: signal receiver, allosteric transactivator, and adaptor for downstream pathways. In that regard, a recently published paper reported an unbiased genetic screen of ErbB3 mutants revealing 18 ErbB3 mutants that enhanced the oncogenic potential by stimulating its catalytically competent ErbB2 partner through enhanced dimerization [77]. Given that across all cancers, somatic *ERBB3* mutations occur at a frequency of 2.1%, these findings support a rationale for treating cancers with these alterations with kinase inhibitors and/or therapeutic antibodies targeting ErbB2/ErbB3. In addition, this and many other studies on RTKs suggest that we need to consider alterations in these enzymes not only in terms of their impact on catalysis but also for their impact on their adaptor, scaffolding, and allosteric transactivation functions [74].

### 5.2. The Tyrosine Phosphatase SHP2 Exerts Signaling Functions Independent of Its Catalytic Activity

The SH2-containing tyrosine phosphatase SHP2, the product of the *PTPN11* proto-oncogene, represents a key signaling protein and proto-oncogene product in the RAS/ERK pathway. SHP2 consists of two N-terminally located SH2 domains arranged in tandem (N-SH2 and C-SH2), followed by the catalytic domain and a C-terminal tail containing a regulatory bipartite tyrosine phosphorylation motif, implicated in sustaining SHP2 activation [78,79]. In its inactive state, SHP2 resides in a closed autoinhibited conformation stabilized by the binding of the tandem SH2 domain to the catalytic domain. In the presence of tyrosine-phosphorylated proteins, e.g., tails of RTKs, the tandem SH2 domain will be displaced from the catalytic domain, thereby allowing its access to substrates [78]. The majority of these substrates remain ill-defined [80], and its positive influence on RAS/ERK pathway activation appears to be manifold, including the dephosphorylation of a tyrosine residue in RAS, which enhances RAF recruitment in its unmodified state [81]. This key function of SHP2 is also reflected by its frequent dysregulation in cancer, either resulting from overexpression of its activators such as docking/adapter proteins of the GAB and FRS families [65] or from the amplification or gain-of-function mutations of *PTPN11* itself [82]. Interestingly, *PTPN11* germ-line mutations are found in two syndromes of the RASopathy complex [83], Noonan and LEOPARD syndrome. Interestingly, while gain-of-function mutations in SHP2 dominate the spectrum in Noonan syndrome patients, dominant-negative, loss-of-function mutations abrogating phosphatase activity occur in LEOPARD syndrome patients and rather seem to divert RTK derived signals into the PI3K axis and highlight the adaptor functions of this phosphatase [65]. As RAS-driven cancers were considered undruggable until recently and still represent a clinical challenge, the discovery that some oncogenic RAS mutants that still undergo a GTP cycle require SHP2 for GTP loading spurred the development of SHP2 inhibitors. In 2018, a series of publications reported in various preclinical models for KRAS-driven carcinoma entities that SHP2 inhibition augments the antitumor effects of MEK inhibitors [84]. However, these respective allosteric inhibitors are unlikely to eliminate all functions of this phosphatase, which have emerged early on in this research field and also relate to functions outside of the RAS/ERK axis [85], including the suppression of p53 in a LEOPARD syndrome zebrafish model [86]. Moreover, there are recent data suggesting that the tandem SH2 domains can protect phosphotyrosine residues in SHP2 interaction partners such as GAB docking proteins [87]. The further characterization of phosphatase-independent functions of SHP2 represents an interesting area for future research, in particular as SHP2 with its pleiotropic and still enigmatic functions represents a key signaling element not only in the cancer cell compartment but also in the tumor microenvironment, including in its diverse set of infiltrating immune cells [87] (Table 1). Of note, the SH2 domains of SHP2 have been recently implicated in phase separation events in fibroblast growth factor receptor (FGFR) signaling [88].

### 5.3. Rapidly Accelerated Fibrosarcoma (RAF) and Kinase Suppressor of RAS (KSR) Proteins: Moonlighting and Allostery

Non-enzymatic functions of RAS/ERK pathway components also prominently feature on the tier of RAF kinases. In mammals, this small family of Ser/Thr-kinases consists of the ARAF, BRAF, and RAF1 isoforms, which, despite the fact that they can be all activated by RAS and all phosphorylate MEK, differ in their regulation by post-translational modifications, their interactome and their intrinsic kinase activity [66,89]. BRAF contains the highest intrinsic kinase activity followed by RAF1 and ARAF. For RAF1 (also known as CRAF), several so-called non-catalytic “moonlighting” functions have been described that are unrelated to its role in the RAS/ERK pathway [90] (Table 1), such as its influence on STAT3 signaling [91], the HIPPO/YAP/TAZ pathway by sequestering its gatekeeper kinase mammalian STE20-like protein kinase 2 (MST2) [92,93], by blocking cell death by binding to ASK1 [94] or by controlling cell migration through its binding to RHO-associated protein kinase 1α (ROK1α) [95]. Indeed, these kinase-independent functions of RAF1 are strongly supported by in vivo experiments involving two distinct knock-in alleles encoding truly kinase-inactive RAF1 proteins and failed to recapitulate the protective effect of the loss of RAF1 expression in a lung cancer model [96].

The KSR proteins represent close relatives of the RAF proteins and are now considered as pseudokinases serving as a scaffold for the entire RAF/MEK/ERK module. Interestingly this scaffolding function is even required for maintaining the transformed phenotype of melanoma cells driven by BRAF^V600E^, an oncoprotein that is otherwise exempted from many regulatory requirements such as activated RAS [97,98,99]. In addition, KSR proteins have more recently been recognized as important allosteric activators of BRAF (Figure 4), involving an evolutionary conserved dimer interface (DIF) in the (pseudo)kinase domains of these proteins [100,101] (Table 1). The pairing between BRAF and KSR1 is further modulated by 14-3-3 dimers serving as “matchmakers” at the C-termini of both proteins and in particular by a salt-bridge formed between two helices at their N-termini [102].

**Table 1:** Comparison of the “Role as enzyme” and “Role as adapter” for the main proteins discussed in this review.

Protein	Role as Enzyme	Role as Adapter	Ref.
BTK	Phosphorylation/activation of PLCγ	Tumor suppressor function Membrane recruitment of PIP5Ks causing PI45P_2_ production	[16,17,18]
ITK	Phosphorylation/activation of PLCγ	TCR/CD3-triggered actin polymerization	[30]
PI3K (p110γ)	Phosphorylation of PI45P_2_ to yield PIP_3_ Regulation of leukocyte migration and inflammation	Constitutive interaction with PDE3B and promotion of PDE3B activity (cAMP hydrolysis) in cardiomyocytes	[34,37]
SHIP1	Hydrolysis of PIP_3_ to yield PI34P_2_ Inhibition of PKB activation and Ca^2+^ mobilization upon BCR-FcγRIIB crosslinking	Attenuation of RAS activation by GRB2-SOS competition Inhibition of RAS by DOK1-RASGAP1 recruitment Attenuation of NOD2-induced NFκB activation by interacting with XIAP	[38,44,45,47,49,50]
HER3/ ErbB3	Naturally inactive kinase (pseudokinase) or kinase with low intrinsic enzymatic activity	Allosteric transactivator of catalytically competent EGFR family members, most notably HER2/ErbB2; adaptor, phospho-tyrosine residues as docking sites for PI3K recruitment	[73,74,76]
SHP2	Protein tyrosine phosphatase	Protection of phosphotyrosine residues by tandem SH2 domain against dephosphorylation	[87]
RAF1	Protein serine/threonine kinase	Various adaptor functions, see text for details	[90,91,92,93,94,95]
KSR1	Naturally inactive kinase (pseudokinase) or kinase with low intrinsic enzymatic activity	Scaffolding functions for the RAF/MEK/ERK pathway; allosteric transactivator for BRAF	[100]

However, their status as truly inactive pseudokinases, which is strongly supported by the absence of a key catalytic lysine residue conserved across most eukaryotic protein kinases [101], has been recurrently challenged over the years. A recent study suggests that at least ATP binding, not necessarily hydrolysis and transfer of the gamma-phosphate, is required for the allosteric transactivation potential of KSR1 [103]. However, no matter whether KSR proteins represent real pseudokinases or reactivate residual intrinsic kinase activity under certain circumstances promoting a catalytically competent kinase domain fold, there is clear biochemical and genetic evidence that truly kinase-dead cancer-associated BRAF mutants, such as BRAF^D594G^ lacking the catalytic aspartate of the DFG-motif, activate the MEK/ERK pathway by acting as dimerization partners of catalytically competent RAF proteins [104,105]. Biochemical studies showed that this paradoxical behavior of kinase-dead BRAF oncoproteins requires two structural prerequisites. First, both kinase-dead BRAF and its dimerization partner must be able to interact with activated RAS (RAS-GTP), as it promotes their membrane recruitment and subsequent dimerization. Therefore, and in sharp contrast to the RAS-independent BRAF^V600E^ mutant, kinase-dead BRAF mutants frequently co-occur with RAS oncoproteins and disease modelling in genetically engineered mouse models (GEMMs) confirmed the suspected cooperativity between both oncoproteins emerging from statistical associations observed in human cancers and biochemical experiments in tissue culture [104,105]. Alternatively, RAS-GTP can be supplied by other means such as aberrant RTK signaling or loss of RAS-GAPs such as NF1 and thereby promotes the oncogenic activity of kinase-dead BRAF mutants [106,107,108]. Second, this paradoxical activation requires the presence of the evolutionary conserved DIF residue R509, by which kinase-dead BRAF protomer allosterically transactivates the still inactive receiver protomer by switching the latter into an active conformation [67,68,99]. In that regard, kinase-dead BRAF oncoproteins behave similarly to KSR proteins and also imitate BRAF molecules occupied by ATP-competitive inhibitors, which can, as discussed in the next paragraph, “Pharmacological implications”, also cause a similar paradoxical ERK pathway activation.

## 6. Pharmacological Implications

The often-overlooked adaptor and scaffolding functions of enzymes are also highly relevant for our understanding of drug action or failure. Most targeted therapy compounds act via the competitive or non-competitive principle and thereby block the catalytic action of the enzyme by competing with its (co-)substrates, such as ATP. Indeed, famous protein kinase inhibitors such as the oncoprotein BCR::ABL1 targeting imatinib or the BRAF inhibitor vemurafenib act as ATP competitive drugs, thereby not affecting the expression level of their targets [109,110]. Likewise, allosteric inhibitors like the fourth generation BCR::ABL1 inhibitor Asciminib (aka ABL001) or the SHP2 inhibitor SHP099 do not remove their target, although they can serve as a “molecular glue” stabilizing inactive conformations and thereby might impinge on certain protein–protein interactions, e.g., those mediated between the tandem SH2 domains of SHP2 and the cytoplasmic tails of RTKs [111,112]. Alternatively, an inhibitor might eliminate the kinase function but at the same time increase the non-catalytic role of the intended drug target. For example, small molecule inhibitors targeting ERK5, which serves not only as a kinase but also as a transcriptional regulator, enhance the latter functions by stimulating the nuclear localization of this large MAPK and the exposure of its transcription promoting moiety [113,114].

That the effects of an inhibited enzyme do not always mirror the same “phenotype” as its absence is a well-established but often ignored fact in the fields of signal transduction and targeted therapies. In fact, reviewers often would like to see that the outcomes of “pharmacological” experiments are corroborated by genetic approaches, for example by RNAi-induced mRNA depletion or CRISPR/Cas9-mediated gene editing. These are very valid suggestions that can rule out or identify potential off-target effects and confirm proposed mechanisms of action. For example, the allele-specific knockdown of BRAF^V600E^ phenocopies the re-differentiation effect of the vemurafenib tool compound PLX4720 in human colorectal cancer cell lines, thereby showing that lack of this oncogenic kinase induces a similar phenotype as its blockade [115]. BRAF, however, is also a prime example where the kinase occupied by an ATP competitive inhibitor is causing a paradoxical effect under certain circumstances. This lesson was learnt shortly after the first melanoma patients treated with BRAF^V600E^ selective inhibitors developed secondary neoplasia, in most cases “white” skin tumors such as keratoacanthoma, but in rare cases also leukemia or pancreatic cancers [70,109,116,117,118]. Subsequent analyses showed that these secondary neoplasms were either driven by too much RAS activity induced by upstream signals, either supplied by RAS mutations or elevated RAS-GTP loading by upstream signals. This in turn promotes dimerization of drug-bound BRAF with drug-free RAF proteins and allosteric transactivation of the latter, in a similar way as described above for kinase-dead BRAF mutants as structure function analyses revealed, in particular those addressing the RAS-binding domain and the DIF in full-length RAF proteins [99,104]. Moreover, due to negative allostery, the drug-bound RAF protomer forces the drug-free dimerization partner into an active conformation that precludes drug accommodation but allows or even enhances catalysis. As a result, the drug-free RAF protomer will remain activated and drive ERK pathway activation [69]. Importantly, this drug-induced complication does not occur in cells expressing BRAF^V600E^, as tumors driven by this oncoprotein, at least in a treatment-naïve state, often contain only low levels of RAS-GTP, and BRAF^V600E^ selective inhibitors intercept with their target while it is in a monomeric state [69,119].

These insights, which were obtained in the 2010s following the introduction of BRAF inhibitors into the clinic [69,109], taught the targeted therapy field several important lessons. Regarding the first lesson, we would like to quote John Donne´s famous phrase “No man is an island entire of itself”, which certainly is true for proteins as well. Proteins usually are not acting as mavericks but in well-organized assemblies/complexes. For practical reasons, however, kinase inhibitor development usually starts with screening of compounds against a panel of purified recombinant kinases, which are sometimes not even representing the full-length protein [120,121], or rely on a combination of structural biochemistry/bioinformatics of purified kinase domains [109]. In order to ensure that the activity of the kinase of interest and not of a contaminating or co-precipitated one is measured, these approaches are, as informative and successful as they have been, very reductionistic. The kinase is “stripped bare” by stringent lysis buffers, the in vitro kinase reaction takes place in an aqueous solution with little similarity to the crowded cytoplasm and the lack of interaction partners and post-translational modifications driven by the “social network” of the kinase might affect its enzymatics. For example, critical interaction partners controlling its conformation or quaternary relationships, which could affect substrate choice, might be simply missing. Based on these reductionist systems, the critical homo- and heterodimerization of BRAF that underlies the paradoxical action of BRAF^V600E^ selective compounds was missed and only discovered retrospectively.

Second, as the aforementioned example of negative allostery highlights, binding of ATP competitive drugs dynamically affects target protein structure and vice versa. Indeed, protein kinase inhibitors can be grouped into compounds with higher binding preference to an active or inactive conformation of kinase domains [119,122]. Specifically, type I and type II inhibitors can be distinguished based on their binding modes in which the relative orientation of the αC-helix, a key region in kinase regulation [123], and the DFG-motif at the N-terminal border of the activation loop to each other is used as a criterion [124]. Type I inhibitors, such as the BCR::ABL/SRC inhibitor dasatinib, stabilize the kinase domain in its active “αC-helix-in/DFG-in” conformation, while type II compounds such as sorafenib stabilize the “αC-helix-in/DFG-out” conformation [122,125]. The clinically applied BRAF^V600E^ specific drugs vemurafenib, dabrafenib, and encorafenib represent so-called I^1/2^ inhibitors inducing a “αC-helix-out/DFG-in” conformation with special intermediate features [124]. Interestingly, these type I^1/2^ compounds bind to BRAF^V600E^ in its monomeric state, while type II drugs are not subject to negative allostery and bind to RAF dimers very efficiently and even stabilize them in such a way that these chemicals can be used as positive controls in experiments assessing RAF dimerization [102]. As a consequence, these drugs can have profound consequences on the size and composition of RAF-containing multiprotein complexes [126], and it can be assumed that RAF inhibitors and their targets are no exemption. Therefore, it will be of particular interest to study the effects of kinase inhibitors on the protein–protein interaction repertoire and potentially adapter functions of their targets.

Third, the almost 15 years of trials and tribulations with RAF inhibitors also emphasize that medicine would probably benefit from so-called type IV compounds that bind to their target in an allosteric manner outside of the ATP binding pocket [122] and two recent independent clinical studies using peptides targeting the DIF of BRAF demonstrate the general feasibility of this approach [127,128]. Moreover, the recent advances in developing proteolysis-targeting chimera (or PROTAC), hetero-bifunctional small molecules that bind with a so-called “war-head”, often derived from an established kinase inhibitor, to their target and recruit at the same time an E3 ubiquitin ligase, represents a promising area of drug development as it would eliminate both the catalytic activity and the adapter functions of an enzyme. Indeed, recent studies demonstrated that PROTACs bypass the aforementioned limitations of ATP competitive RAF inhibitors arising from drug-induced dimerization and were even able to promote degradation of kinase-dead BRAF oncoproteins, suggesting that the PROTAC approach can be also leveraged to target other pseudokinases with important adaptor/scaffolding functions discussed in this review such as HER3 [129,130].

Lastly, the studies discussed in this paragraph illustrate the need for basic research into the intricacies and complexities of established and emerging enzymatic drug targets. We need a comprehensive understanding of both their enzymatic and all other functions. When and where are they interacting with whom in the cell? And how are these often-dynamic interactions affected by drugs? To answer these questions, complementary and multidisciplinary approaches will be required, including proteomics and standard protein biochemistry, but also more modern approaches revealing the dynamics of protein–protein interactions in living cells such as split luciferase complementation [102,120] and proximity biotinylation approaches [131]. In addition, the comparison of knock-out vs. knock-in alleles in model organisms continues to provide valuable insights into the (non)catalytic functions of enzymes, as it has been demonstrated by the analysis of knock-out alleles for *Braf* and *Raf1* [132,133,134] and of various knock-in alleles preventing the activation of their gene products by removing activating phosphorylation sites [135,136] or by eliminating kinase activity altogether by mutating key catalytic residues [96,105,137] (also see chapters on BTK and PI3K^p110γ^). In that regard, Kung and Jura (2016) provide a comprehensive catalogue of examples across the kinome in which knock-out and kinase-inactivating knock-in phenotypes do not match [74]. The spectrum of the phenotypes of these allele series illustrate which protein functions are related to enzymatic or scaffolding functions. Likewise, studying naturally occurring alleles eliminating enzymatic functions such as in the LEOPARD syndrome-associated SHP2 mutants in human conditions [86] or the analysis of pseudoenzymes [60], also within their evolutionary context [101], will reveal additional insights regarding how enzymes adopted adaptor functions or how adaptors acquired enzymatic functions. With the advances in gene editing using CRISPR/Cas approaches, such studies are no longer limited to animal models, but can nowadays be extended to human cell line models as well.

## 7. Conclusions

In this review, we focused on the non-catalytic properties of some kinases such as BTK, PI3K, and RAF1 as well as phosphatases such as SHIP1 and SHP2 to highlight the corresponding non-catalytic adapter functions of these signaling proteins (Table 1). The experimental analysis of such dual functions of kinases and phosphatases is not only a very exciting, demanding academic exercise, but also enables a better understanding of reasons why certain pharmacological inhibitors either do not perform in the expected and desired way or even, which can be extremely harmful, particularly in the context of malignant diseases, cause activation of a kinase or of another enzyme that interacts with this kinase (“paradoxical activation”). Current pharmaceutical developments (e.g., PROTACs) can help minimize respective problems of inhibitors of catalytic functions. It is therefore still extremely important and necessary to molecularly, structurally, and functionally investigate the intramolecular and intermolecular interactions of the various domains of a signaling enzyme in order to advance pharmacological developments that provide maximum benefit for patients.

## Supplementary Materials

The following supporting information can be downloaded at: https://www.mdpi.com/article/10.3390/cells13151249/s1, Table S1: Abbreviations.
