# Supplementary material for: Enzyme Is the Name—Adapter Is the Game
Source: Cells. 2024 Jul 25;13(15):1249. doi: 10.3390/cells13151249 (PMC11311582; doi:10.3390/cells13151249)
Supplement: Supplementary file 1 [file cells-13-01249-s001.zip › Table S1. Abbreviations.pdf]

Protein	Extended name	Function
ARAF	Rapidly accelerated fibrosarcoma A	Ser-/Thr-kinase
ASK1	Apoptosis signal-regulating kinase 1	Ser-/Thr-kinase
BAM32	B cell adaptor molecule 32 kDa	Adapter protein
BCR	B cell receptor	Immuno receptor
BCR::ABL1	Breakpoint cluster region::Abelson kinase	Tyrosine kinase
BLNK	B cell linker protein (SLP65)	Adapter protein
BRAF	Rapidly accelerated fibrosarcoma B	Ser-/Thr-kinase
BTK	Bruton´s tyrosine kinase	Tyrosine kinase
CD3	Cluster of differentiation 3	TCR signal generation
CIN85	Cbl-interacting 85 kDa	Adapter protein
Cas9	CRISPR-associated protein 9	Nuclease
DOK1	Downstream of kinase 1	Adapter protein
EGFR	Epidermal growth factor receptor	Tyrosine kinase
ErbB3	Receptor tyrosine kinase ErbB3	Kinase-impaired receptor
ERK	Extracellular signal-regulated kiinase	Ser-/Thr-kinase
FcγRIIB (CD32B)	Inhibitory IgG receptor, low-affinity	Immunoglobulin receptor
GAB	GRB2-associated binder	Adapter protein
GRB2	Growth factor receptor-bound protein 2	Adapter protein
HCK	Hematopoietic cell kinase	Tyrosine kinase
HER3	Human epidermal growth factor receptor 3	Kinase-impaired receptor
ITK	Interleukin-2-inducible T-cell kinase	Tyrosine kinase
JAK	Janus kinase	Tyrosine kinase
KSR1	Kinase suppressor of RAS 1	Adapter protein
MAPK	Mitogen-activated protein kinase (ERK)	Ser-/Thr-kinase
MEK	Mitogen-activated protein kinase kinase	Dual-specificity kinase
MEKK	Mitogen-activated protein kinase kinase kinase	Ser-/Thr-kinase
MST2	Mammalian STE20-like protein kinase 2	Ser-/Thr-kinase
NFκB	Nuclear factor kappa B	Transcription factor
NOD2	Nucleotide binding and oligomerization domain 2	Innate immunoreceptor
PDE3B	Phosphodiesterase 3B	Phosphodiesterase
PI3K	Phosphatidylinositol 3-kinase	lipid kinase
PIM	Proviral integrations of Moloney virus	Ser-/Thr-kinase
PKA	Protein kinase A	Ser-/Thr-kinase
PKB	Protein kinase B	Ser-/Thr-kinase
PKC	Protein kinase C	Ser-/Thr-kinase
PLC	Phospholipase C	Phospholipase
PTEN	Phosphatase and tensin homolog deleted on chrom. 10	Lipid phosphatase
RAF	Rapidly accelerated fibrosarcoma	Ser-/Thr-kinase
RAF1	Rapidly accelerated fibrosarcoma 1	Ser-/Thr-kinase
RAS	Rat sarcoma	Small G-protein/GTPase
RASGAP1	RAS GTPase-activating protein	GTPase-activating protein
RIP2	Receptor-interacting protein kinase	Ser-/Thr-kinase
ROK	Rho-associated protein kinase	Ser-/Thr-kinase
RTK	Receptor tyrosine kinase	Tyrosine kinase
SHC	SRC homologous and collagen	Adapter protein
SHIP1	SH2-containing inositol phosphatase 1	Lipid phosphatase
SLP65	SH2-containing linker protein 65 kDa (BLNK)	Adapter protein
SOS	Son of sevenless	Guanine nucleotide exchange factor
SRC	Sarcoma	Tyrosine kinase
TAPP2	Tandem PH domain-containing protein 2	Adapter protein
TCR	T cell receptor	Immuno receptor
TEC	TEC protein tyrosine kinase	Tyrosine knnase
XIAP	X-linked inhibitor of apoptosis protein	E3 ubiquitin protein ligase
